# Exciting experiences make neurons less excitable

**DOI:** 10.7554/eLife.29763

**Published:** 2017-07-27

**Authors:** Prakruti Nanda, Tal Inbar, Joseph F Bergan

**Affiliations:** 1 Neuroscience and Behavior Program and the Department of Psychological and Brain Sciences, University of Massachusetts Amherst, Amherst, United States; 1 Neuroscience and Behavior Program and the Department of Psychological and Brain Sciences, University of Massachusetts Amherst, Amherst, United States; 1 Neuroscience and Behavior Program and the Department of Psychological and Brain Sciences, University of Massachusetts Amherst, Amherst, United Statesjbergan@umass.edu

**Keywords:** olfactory, pheromone, learning, electrophysiology, social, Mouse

## Abstract

Neurons in the brain of a female mouse that respond to the scent of a given male become suppressed after mating.

**Related research article** Gao Y, Budlong C, Durlacher E, Davison IG. 2017. Neural mechanisms of social learning in the female mouse. *eLife* **6**:e25421. doi: 10.7554/eLife.25421

Animals often need to remember their past encounters with other members of the same species. For many animals, including mice, smell plays a critical role in forming these social memories throughout their entire life (Keller et al., 2009). For example, a mouse pup must remember its mother’s scent (Sullivan et al., 1990), a juvenile mouse must learn the odors of its playmates (Smith, 1982), and an adult mouse needs to identify its offspring, social peers or mates (Dulac et al., 2014; Ben-Shaul et al., 2010; Bruce and Parrott, 1960).

Mice detect the scent of other mice partly via their vomeronasal organ, which is found between the nose and mouth (Halpern and Martínez-Marcos, 2003). The sensory neurons in this organ send signals to a part of the brain called the accessory olfactory bulb, which is in turn connected to the centers of the brain that influence social behaviors like aggression, parenting and mating (Choi et al., 2005). The neural circuits that trigger specific behaviors in response to odors are not exclusively hardwired. Rather, the function of these circuits is shaped by social experience; this is an example of a wider phenomenon referred to as neural plasticity (Shea et al., 2008; Citri and Malenka, 2008).

Now, in eLife, Ian Davison and colleagues – including Yuan Gao as the first author – report on a new form of neural plasticity seen in the accessory olfactory bulb of female mice (Gao et al., 2017). The study used mice in which recently activated neurons produce green fluorescent protein (GFP) and can be easily identified by a green glow. Gao et al. – who are based at Boston University and Mount Holyoke College – took female mice and introduced them to a male mouse. Some of the females were also allowed to mate with the male and some were not (Figure 1). Gao et al. then collected slices from the brains of the female mice and recorded how different neurons in the slices responded to electrical stimulation.

**Figure 1.**
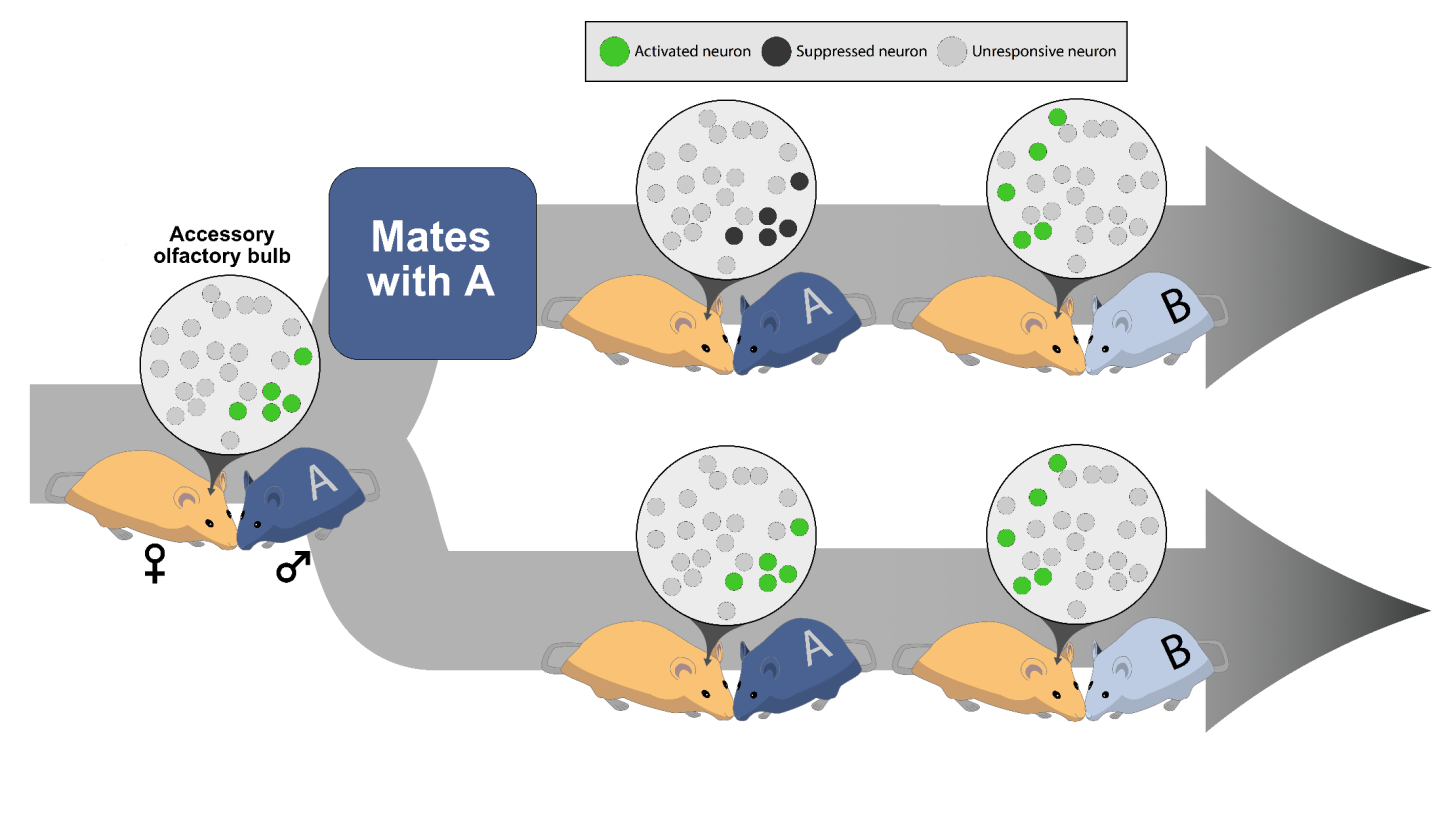
Social experience alters neural plasticity within the accessory olfactory bulb. The odor of a male (♂) mouse activates a subset of neurons in the accessory olfactory bulb of a female (♀) mouse’s brain. The green circles in the inset represent the neurons that have been activated; the light gray circles indicate neurons that are not involved in a particular interaction. (Top) If the female mates with the male, the neurons activated by that male’s odor become suppressed when she encounters the same male (labeled A) again; dark gray circles represent neurons suppressed during a repeated sensory experience. However, if the female encounters a new male (labeled B), a different set of neurons can still become activated. (Bottom) If the female does not mate with male A, the neurons in her accessory olfactory bulb continue to respond if she encounters the same male (male A) again or a different male mouse (male B).

The experiments showed that, at first, recently active neurons (that is, those with GFP) were as excitable as the inactive or unresponsive neurons (those without GFP). This was true for the female mice who had mated and those that had not. However, GFP-positive neurons from mated females became dramatically less excitable when the stimulation was repeated, while the GFP-negative neurons remained largely unaffected. This difference was not seen in the unmated females, and indicates that mating triggers a change that makes activated neurons in the accessory olfactory bulb become less excitable in the future.

Next, Gao et al. compared the responses from two different kinds of neuron within the accessory olfactory bulb: mitral cells and granule cells. The mitral cells receive signals from sensory neurons and send signals to other centers of the brain, while the granule cells inhibit the mitral cells. Gao et al. saw that all the mitral cells were inhibited more after mating, and so inhibition could not explain why only the GFP-positive mitral cells became less excitable after mating. Changes in the strength of connections between the mitral cells and other neurons could also not explain the difference. Instead, it appears that something within the recently active mitral cells themselves changed to make these cells less excitable. This has not been seen before, and so represents a new way to store social memories. Nevertheless, these findings are still consistent with the idea that social memories are formed in areas of the brain that are among the first to process sensory information (Wilson et al., 1987).

Gao et al. reason that this new plasticity mechanism likely helps female rodents to recognize and remember their mating partners (Figure 1). After successfully mating with a male, the mitral cells in the accessory olfactory bulb that respond to the male's scent are all suppressed. This likely stops that male’s scent from triggering responses in other centers of the brain, while still allowing the female to identify, and respond to, a new male. For example, the neural plasticity described by Gao et al. may contribute to the 'Bruce effect' in which the presence of an unfamiliar male causes a female mouse to terminate her pregnancy, while the presence of the father does not (Bruce and Parrott, 1960).

This new kind of neural plasticity underscores the role of the accessory olfactory bulb in allowing animals to effectively recognize others and to form social memories. It also highlights the differences in how the social brain processes a familiar versus an unfamiliar animal. It is possible, however, that the excitability of mitral cells in the accessory olfactory bulb is also suppressed by other experiences, such as defending against a predator or caring for pups. Alternatively, animals may adapt to the sensory cues of a familiar mate while maintaining a strong response to dangerous predators. It will be interesting to learn from future experiments whether the plasticity described by Gao et al. is specific to mating or, rather, if it represents a more general mechanism for social learning in the accessory olfactory bulb.
